# Au Nanoparticles-Doped Polymer All-Optical Switches Based on Photothermal Effects

**DOI:** 10.3390/polym12091960

**Published:** 2020-08-29

**Authors:** Yue Cao, Daming Zhang, Yue Yang, Baizhu Lin, Jiawen Lv, Fei Wang, Xianwang Yang, Yunji Yi

**Affiliations:** State Key Laboratory of Integrated Optoelectronics, College of Electronic Science and Engineering, Jilin University, Changchun 130012, China; yuecao17@mails.jlu.edu.cn (Y.C.); zhangdm@jlu.edu.cn (D.Z.); a2604702999@163.com (Y.Y.); linbz17@mails.jlu.edu.cn (B.L.); lvjw18@mails.jlu.edu.cn (J.L.); wang_fei@jlu.edu.cn (F.W.); yangxw1918@mails.jlu.edu.cn (X.Y.)

**Keywords:** all-optical switches, Au nanoparticles, polymer nanocomposite materials, photothermal effects, mode switches, all-optical networks, wearable technology, flexibility device

## Abstract

This article demonstrated the Au nanoparticles-doped polymer all-optical switches based on photothermal effects. The Au nanoparticles have a strong photothermal effect, which would generate the inhomogeneous thermal field distributions in the waveguide under the laser irradiation. Meanwhile, the polymer materials have the characteristics of good compatibility with photothermal materials, low cost, high thermo-optical coefficient and flexibility. Therefore, the Au nanoparticles-doped polymer material can be applied in optically controlled optical switches with low power consumption, small device dimension and high integration. Moreover, the end-pumping method has a higher optical excitation efficiency, which can further reduce the power consumption of the device. Two kinds of all-optical switching devices have been designed including a base mode switch and a first-order mode switch. For the base mode switch, the power consumption and the rise/fall time were 2.05 mW and 17.3/106.9 μs, respectively at the wavelength of 650 nm. For the first-order mode switch, the power consumption and the rise/fall time were 0.5 mW and 10.2/74.9 μs, respectively at the wavelength of 532 nm. This all-optical switching device has the potential applications in all-optical networks, flexibility device and wearable technology fields.

## 1. Introduction

Polymer materials are widely used in the field of thermal optical switches due to the characteristics of low cost, high thermo-optical coefficient and flexibility [1,2,3]. Currently, realizing the optically controlled devices with low optical losses, low power consumption and fast response time are becoming the tendency and research highlight because of the application of the optical switches in all optical networks [4,5,6,7].

To improve these performances of the device, various nanocomposite materials of polymer-doping functional materials have been studied such as the erbium-doped material, Nd-doped polymer, NaYF_4_: Yb^3+^, Er^3+^ doped polymer and graphene doped polymer material [8,9,10,11]. In 2018, Xing proposed a NaYF4: 18% Yb^3+^, 2% Er^3+^ doped polymer thermal optical (TO) switch, realizing the loss compensation in the waveguide. The electrode-driving power and the loss compensation were 7 mW and 3.8 dB, respectively. In 2019, Cao proposed a monolayer graphene doped polymer TO switch with low power consumption and fast response time. The power consumption and rise/fall time are 7.68 mW and 40/80 μs, respectively. At present, these nanocomposite materials are mainly used in the field of optical amplifiers or electrode-driving optical switches.

However, for the optically controlled optical switches, they are mainly achieved by saturated absorption [12], Kerr effect [13,14] and photothermal effect [15,16,17]. For the modulation method of saturation absorption, a large modulation depth will cause a large loss of the device [18]. For the optical switches based on the Kerr effect, the weak nonlinearity of the conventional waveguide materials requires a high pump power and large waveguide dimension, which are not conducive to device integration. Recently, the all-optical switches based on the photothermal effect have been applied to realize a small device dimension and high integration of the device [19].

To realize the all-optical switch based photothermal effects, the polymer-doping functional materials could be applied in the devices. However, there are three crucial factors limiting the performance of the device: photothermal properties of the materials, compatibility with devices and the pumping method. First, The photothermal materials such as semiconductors, graphene, noble metals and metal oxides have been studied in all-optical switch areas [20,21,22]. In 2013, Cameron Horvath reported a graphene–silicon resonant switch increasing the effective index compared to the bare silicon switches [23]. In 2019, Ting Hao proposed a graphene MZI all-optical switch. The pump power was 5.3 mW with a graphene coating of 5 mm long. The rise and fall time was 30 ms and 50 ms, respectively [24]. In 2019, Xinghua Yang realized an all-fiber switch integrated with Au nanorods with a spectral shift efficiency of 0.16 nm/mW [19]. Among these materials, the Au nanoparticles (NPs) could produce a strong electric field under the laser irradiation because of the large electric dipole absorption by surface plasmon polaritons (SPP). The thermal energy would be generated due to the charge carrier transfer inside the NPs. Due to the strong photothermal effects of the Au NPs, the application of the nanocomposite material based on nanogold has great potential in the all-optical switch areas. Second, compared with the inorganic waveguide, polymer materials are of benefit to doping or integration with photothermal materials, which helps compatibility with polymer waveguide. Finally, compared with the side pumping method, the end-pumping method has higher optical excitation efficiency to the photothermal materials, which could reduce the power consumption of the optical switches [25].

In this letter, first, we proposed a base-mode switch based on photothermal effects of Au NPs-doped polymer material. This polymer nanocomposite material was used as the cladding material of the device. The Au NPs were excited by 532 nm pumping light. Due to the strong absorption of Au NPs and the large thermal-optical coefficient of polymer, the refractive index of the polymer nanocomposite material is changed according to the thermal-optical process, realizing the phase shift of the waveguide. Second, we designed a composite first-mode switching device. The Au NPs layer was embedded in the center of the core layer. When the pumping light on, the first-order mode in the waveguide can be controlled by changing the effective refractive index of the first-order mode because of the strong absorption of the base mode at 532 nm pumping light. Meanwhile, the polarization states of the first-order mode in waveguide have also been studied for the integrated all-optical switch device.

## 2. Materials and Methods

### 2.1. Photothermal Effect of the Au Nanoparticles-Doped Polymer Material

When the Au NPs (Abace Biotechnology, Beijing, China) are illuminated, the interaction of light with nano-gold results in the generation of localized surface plasmon resonances (LSPRs). The light gets absorbed at the LSPR frequency and ultimately turned into heat [26,27]. The heat generation depends strongly on the size and shape of the Au NPs.

The efficiency of the absorption processes of Au NPs can be characterized by the absorption cross section (*σ_abs_*). For the spherical Au NPs, the absorption cross section can be analyzed by the Mie analytical model [28,29]. The polarizability of a spherical Au NPs was calculated by the expression:

(1) $$\alpha (\omega )=4\pi R_{\mathrm{Au}}^{3}\frac{\varepsilon_{\mathrm{Au}}-\varepsilon_{p}}{\varepsilon_{\mathrm{Au}}+2\varepsilon_{p}}$$

where *ε_Au_* and *ε_p_* are the relative permittivity of the Au NPs and the polymer material, respectively. *R_Au_* is the nanoparticles radius. *ω* is the angular frequency of the incident light. The absorption cross section of Au NPs is determined by the imaginary part of polarizability, which is given by:

(2) σabs=kImα

where *k* = 2*π*/*λ = nω*/*c* is the wave vector of the incident light. The absorption coefficient of Au NPs is given by [30,31]:

(3) $$A_{\mathrm{abs}}=\frac{\sigma_{\mathrm{abs}}}{S}$$

where *S* is the cross-sectional area of the Au NPs. When the irradiance of incoming light is *I*, the power of heat generation *Q* was calculated by the expression:

(4) $$Q=I\sigma_{\mathrm{abs}}$$

(5) Q=ω8π|3εp2εp+εAu|2ImεAu8πI0cεp=ω8πE02|3εp2εp+εAu|ImεAu

For a single Au NPs, the temperature distribution in the steady-state was calculated by:

(6) $$\Delta T(r)=\frac{V_{Au}Q}{4\pi k_{p}r}$$

where *r* and *V_Au_* are the distance from the center of the Au NPs and the volume, respectively. *k_p_* is the thermal conductivity of the polymer. Due to the large thermal conductivity of Au NPs, the whole nanoparticle will reach the maximum temperature at the same time. The maximum temperature at *r* = *R_Au_* is given by:

(7) ΔTmax=RAu23kpω8π|3εp2εp+εAu|2ImεAu8πI0cεp=RAu23kpω8πE02|3εp2εp+εAu|ImεAu

### 2.2. Devices Design Based on the Au Nanoparticles-doped Polymer Material

According to the strong photothermal effects of the Au NPs, the Au nanoparticles-doped polymer material can be synthesized, and can be applied in the all-optical switch field. Combining the modulation region of the Mach–Zehnder interferometer (MZI) waveguide and the nanocomposite material, the light in the modulation region absorbed by the Au NPs was converted into heat when the pumping light on. Due to the large thermal optical coefficient of the polymer material, the effective refractive index of the optical switches can be easily adjusted, realizing the phase modulation of the optical switches.

Two kinds of all-optical switch structures based on Au nanoparticles-doped polymer material were simulated. One is an inverted ridge waveguide structure with a thin polymer film deposited on the core layer. This polymer film helps reduce the loss of signal light in the waveguide induced by the Au nanoparticles. The cladding is Au NP-doped polymer nanocomposite material, which can be used for signal light adjust after injecting the pumping light. The other is a ridge waveguide structure based on Au NPs-doped polymer material. The nanocomposite material with continuous arrangement Au NPs is embedded in the center of the core layer. When pumping light on, the base mode of the pumping can be absorbed, realizing the control of first-mode with different polarization states in the waveguide.

## 3. Results and Discussion

### 3.1. Single Au NPs Analysis

**Figure 1:**
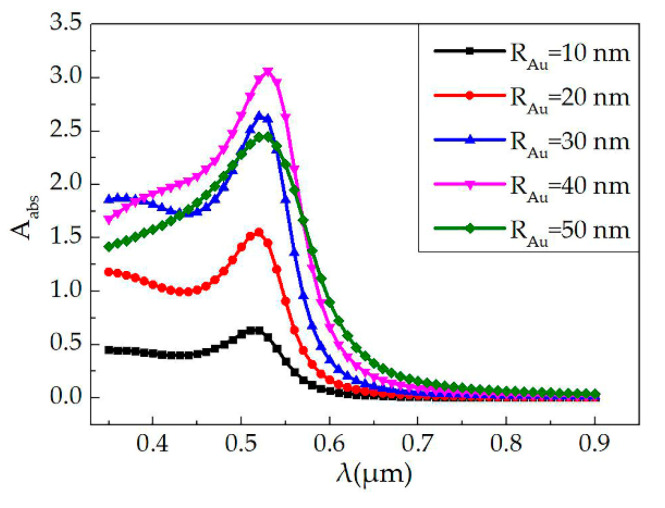
Calculated absorption coefficient of single Au nanoparticles (NPs) materials with different Au NPs radius as a function of wavelength.

The absorption curves of the single Au NPs–polymer nanocomposite illuminated by pumping light were calculated by finite element analysis software. Figure 1 shows the absorption curves of the single Au NPs–polymer nanocomposite material when the wavelength changed from 350 to 900 nm, and the radius of the Au NPs increased from 10 to 50 nm. The curves exhibited a maximum absorption at a wavelength around 530 nm with the Au NPs radius of 40 nm. Due to the increase of the cross-sectional area, a larger radius will lead to a decrease for the absorption coefficient [32,33,34,35,36].

**Figure 2:**
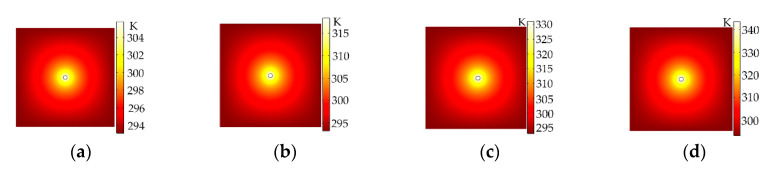
Temperature distributions for single Au NPs–polymer nanocomposites with incoming light power of (**a**) 3000 W/cm^2^; (**b**) 6000 W/cm^2^; (**c**) 9000 W/cm^2^ and (**d**) 12,000 W/cm^2^, respectively *(R_Au_* = 40 nm).

To verify the accuracy of the simulation of the Au NPs photothermal effect by finite element analysis software (COMSOL Inc., Stockholm, Sweden), we calculated the thermal field distributions of the single Au NPs–polymethyl methacrylate (PMMA, Sigma-Aldrich, MO, USA) nanocomposite material with incoming light power of 3000 W/cm^2^, 6000 W/cm^2^, 9000 W/cm^2^ and 12,000 W/cm^2^, respectively (as shown in Figure 2). The thermal conductivity of the PMMA material was 0.19 W/m*K. The calculated maximum temperature at steady state was 305.8 K, 318.4 K, 331.1 K and 343.7 K, respectively. The calculated maximum temperature will increase with the incoming light power increasing when the radius of the Au NPs was much smaller than the incident light wavelength.

### 3.2. All-Optical Switches Based on Au NPs-Doped Polymer

#### 3.2.1. Devices Structure of All-Optical Switches

**Figure 3:**
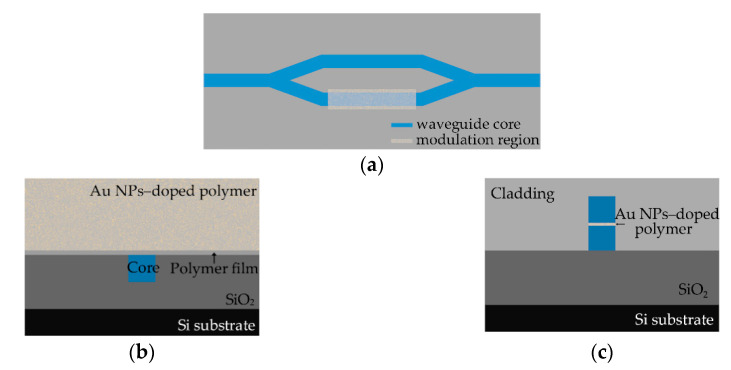
(**a**) Schematic diagram of the Au nanoparticles-doped polymer MZI all-optical switches; (**b**) the cross section of the base-mode switch at wavelength of 650 nm (structure 1: the inverted ridge waveguide structure waveguide with Au NP-doped polymer cladding) and (**c**) the cross section of the first-mode switch at wavelength of 532 nm (structure 2: ridge waveguide structure waveguide with Au NPs is embedded in the center of the core layer).

The all-optical switch structure based on Au nanoparticles-doped polymer material are shown in Figure 3a,b, which shows the inverted ridge all-optical switch structure based on the base mode with a thin polymer film deposited on the core layer (the film thickness (d) ranged from 0.1 to 1 μm). The cladding is Au NP-doped polymer nanocomposite material. a and b are the width and the thickness of the waveguide core, respectively. Figure 3c shows the ridge all-optical switch structure based on the first-mode. The nanocomposite material with continuous arrangement Au NPs was embedded in the center of the core layer.

#### 3.2.2. Mode Characteristics and Light Field Distributions

**Figure 4:**
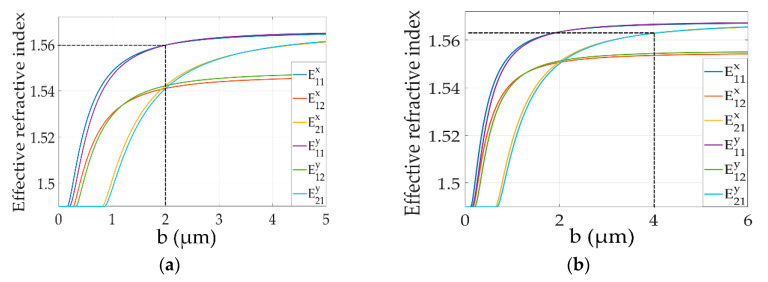
The mode characteristics of the of the Au NPs-doped polymer all-optical switches for (**a**) structure 1 and (**b**) structure 2.

The mode in the waveguide is affected by the size of the core layer and the refractive index of materials using Matlab software (MathWorks.Inc., Natick, MA, USA). Figure 4 shows the curves of the mode characteristics of the all-optical switch in the non-modulated area with the thickness of the core from 0 to 5 μm. The calculated refractive index of the structure 1 at wavelength of 650 nm and the structure 2 at wavelength of 532 nm are shown in Figure 4a,b, respectively. The width of the waveguide core was set as 2 μm. The refractive index of core and cladding was 1.57 and 1.49, respectively.

**Figure 5:**
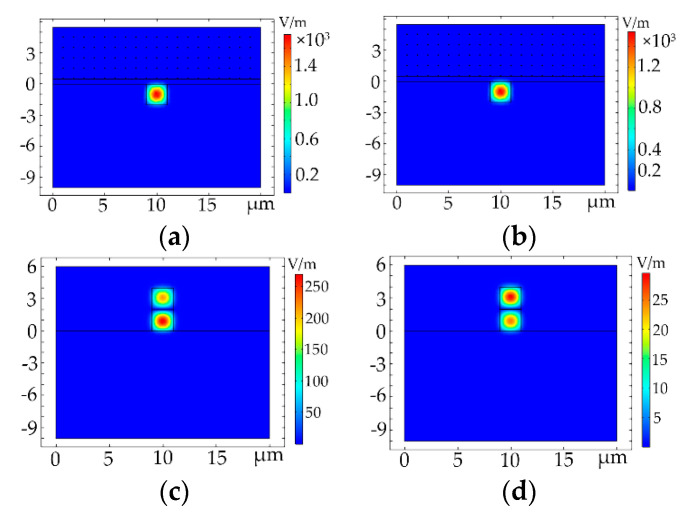
Light field distributions of the Au NPs-doped polymer all-optical switches for (**a**) E_11_ mode (structure 1); (**b**) E_12_ mode (structure 1); (**c**) E_11_ mode (structure 2) and (**d**) E_12_ mode (structure 2).

Figure 5 shows the light field distributions of the Au nanoparticles-doped polymer all-optical switches for structure 1 (*a* = *b* = 2 μm) and structure 2 ((*a* = 2 μm, *b* = 4 μm)), respectively. The base mode (defined as E_11_ mode) and the first-order mode (defined as E_12_ mode) for the optical switch of structure 1 are shown in Figure 5a,b, respectively, and the same for structure 2 are shown in Figure 5b,c, respectively.

#### 3.2.3. Optical Losses Analysis

**Figure 6:**
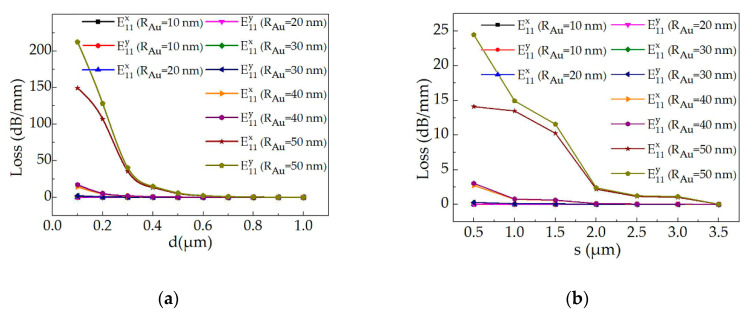
Calculated optical loss of the all-optical switch (structure 1) with the Au radius range from 10 to 50 nm as a function of (**a**) the polymer film thickness (*d*) with Au NPs spacing distance of 1 μm; and (**b**) the Au NPs spacing distance (*s*) with *d* of 0.4 μm (calculated by finite element analysis software).

Figure 6 shows the optical loss induced by Au NPs for structure 1 with polymer film thickness (*d*) from 0.1 to 1 μm, and with a spacing distance of Au NPs uniform dispersed in the polymer material from 0.5 to 3.5 μm. The optical loss of the all-optical switch at wavelength of 650 nm will decrease with the increase of polymer film thickness and the doping ratio. Moreover, the losses of the E^x^_11_ (the TE polarization) mode and the E^y^_11_ mode were calculated with similar values when Au radius lower than 40 nm. The loss of the E^x^_11_ mode and the E^y^_11_ mode was 0.29 dB/mm and 0.33 dB/mm, respectively with *d* of 0.5 μm, *R_Au_* of 40 nm and Au NPs spacing distance of 1 μm (the sectional area ratio of Au NPs was 0.48%).

**Figure 7:**
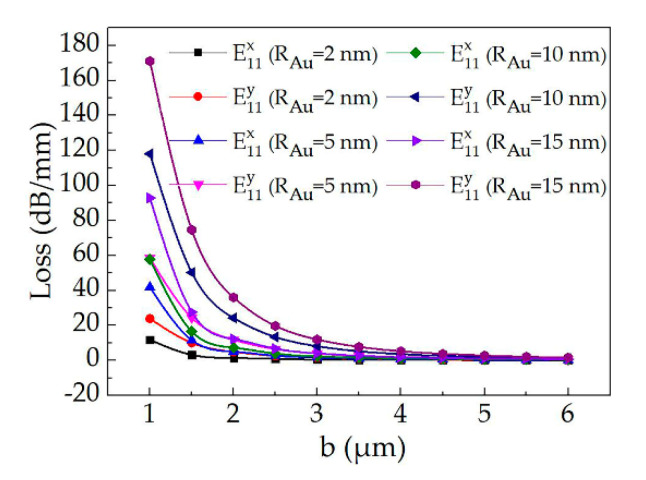
Calculated optical losses of the first-mode switch (structure 2) as a function of the core layer thickness (*b*) with the Au radius of 2 nm, 5 nm, 10 nm and 15 nm, respectively.

For the proposed first-mode switch (structure 2), the optical losses as a function of the core layer thickness (*b*) with the Au radius of 2 nm, 5 nm, 10 nm and 15 nm and the wavelength of 532 nm were calculated (as shown in Figure 7). The losses of the E^x^_12_ mode and the E^y^_12_ mode will decrease with the increase of the core layer thickness. Moreover, the polarization losses were sensitive to the size of Au radius and the core layer thickness. The calculated losses of the E^x^_12_ mode and the E^y^_12_ mode were 1.07 dB/mm and 3.6 dB/mm, respectively with *R_Au_* of 10 nm and *b* of 4 μm. Therefore, by designing the device size and the Au radius, there is only TE polarization mode transmission in the all-optical switch before pumping light on, which the mode switch of E^x^_12_ polarization state can be realized.

#### 3.2.4. Photothermal Analysis of the Proposed Devices

**Figure 8:**
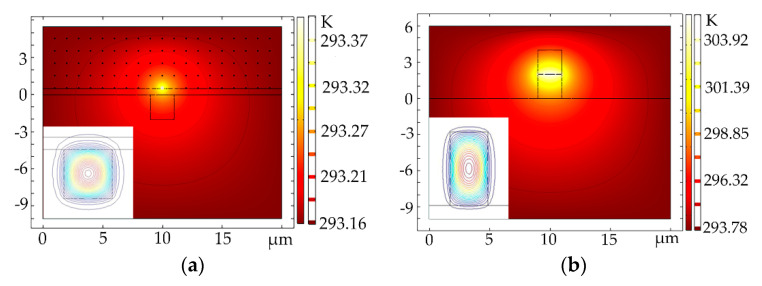
Temperature distributions of the (**a**) structure 1 (*a* = *b* = 2 μm; *d* = 0.5 μm; *R_Au_* = 40 nm) and (**b**) structure 2 ((*a* = 2 μm; *b* = 4 μm; *R_Au_* = 10 nm)) optical switches.

For the proposed devices of Au NPs-doped polymer all-optical switches, the photothermal effect of the Au NPs affected by the light field distributions in the waveguide was simulated. Due to the inhomogeneous distribution of the electric field in the waveguide (the electric field energy in the center of the core layer is the highest, as shown the electric field contours in the inserted picture of Figure 8), the heat generated by Au NPs was inequable at different positions in the waveguide. Figure 8 shows the temperature distributions of the structure 1 and structure 2 optical switches, respectively (multiphysics mode of the finite element analysis method). The results show that the Au NPs would produce higher heat at high electric field energy. The maximum temperature was 293.4 K and 306 K for the structure 1 and structure 2 optical switches, respectively with the incoming light power of 5 μW/μm^2^. It shows that the photothermal conversion efficiency of structure 2 was higher than structure 1 at the same incoming light power.

**Figure 9:**
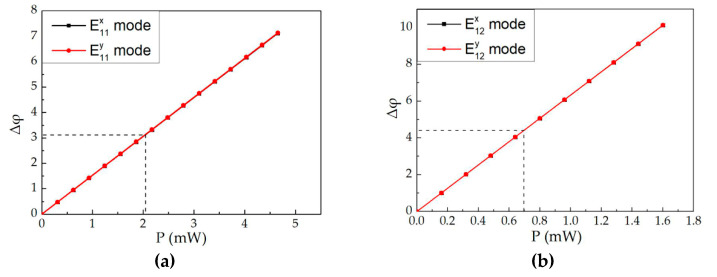
The curves of the calculated the power and the phase change (Δφ) of the all-optical switches for (**a**) structure 1 at wavelength of 650 nm and (**b**) structure 2 at a wavelength of 532 nm.

Figure 9 shows the curves of the relationship between the power consumption (*p*) and the phase change (*Δφ = (2π/λ) × ΔN × L*). *N* and *L* represent the effective refractive index change and the length of the modulation region, respectively. The phase change of the E^x^_11_ mode and the E^y^_11_ mode for structure 1 and the E^x^_12_ mode and the E^y^_12_ mode for structure 2 was linearity in the different incident light power consumption. The calculated power consumption was 2.05 mW and 0.5 mW for structure 1 and structure 2, respectively at *π* of phase shift. The results indicate that the optical switch of structure 2 had lower power consumption due to the higher heat produced by the Au NPs in the waveguide core layer.

**Figure 10:**
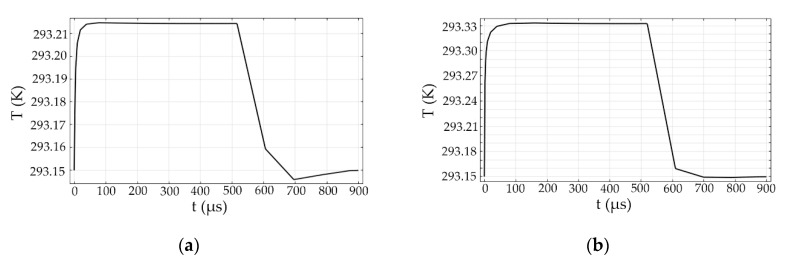
The response time curves of the Au NPs-doped polymer all-optical switches for (**a**) structure 1 (*a* = *b* = 2 μm; *d* = 0.5 μm; *R_Au_* = 40 nm) and (**b**) structure 2 (*a* = 2 μm; *b* = 4 μm; *R_Au_* = 10 nm), respectively.

The response time of the Au NPs-doped polymer all-optical switches for structure 1 with *b* of 2 μm, *d* of 0.5 μm and *R_Au_* of 40 nm and for structure 2 with *b* of 4 μm and *R_Au_* of 10 nm was shown in Figure 10. For structure 1, the calculated rise time and fall time were 17.3 μs and 106.9 μs, respectively, and for structure 2, the calculated rise time and fall time were 10.2 μs and 74.9 μs, respectively.

## 4. Conclusions

In conclusion, we proposed the Au nanoparticles-doped polymer all-optical switches based on photothermal effects. The calculated maximum optical absorption of Au NPs was at a wavelength around 530 nm with the gold radius of 40 nm. There were two kinds of all-optical switches we designed. The first was the base mode switch based on photothermal effects of Au NPs-doped polymer material, which was used as the cladding material of the device. The loss of the E^x^_11_ mode and the E^y^_11_ mode was 0.29 dB/mm and 0.33 dB/mm, respectively with d of 0.5 μm, *R_Au_* of 40 nm and Au NPs spacing distance of 1 μm (the sectional area ratio of Au NPs was 0.48%). The calculated power consumption was 2.05 mW at *π* of the phase shift and wavelength of 650 nm. The calculated rise time and fall time were 17.3 μs and 106.9 μs, respectively. The second was the first-order mode switch based on the E^x^_12_ polarization state. The Au NPs materials were integrated with a waveguide core layer. The calculated losses of E^x^_12_ mode and the E^y^_12_ mode were 1.07 dB/mm and 3.6 dB/mm, respectively with *R_Au_* of 10 nm and *b* of 4 μm. The loss of the E^y^_12_ mode was much larger than E^x^_12_ mode, which the mode switch of E^x^_12_ polarization state could be realized. The calculated power consumption was 0.5 mW for at *π* of the phase shift and a wavelength of 532 nm. The rise time and fall time were 10.2 μs and 74.9 μs, respectively. Compared with the based mode switch, the first-order mode switch had the lower power consumption and fast response time even though it had the larger optical losses.
